# Understanding political priority development for public health issues in Turkey: lessons from tobacco control and road safety

**DOI:** 10.1186/s12961-019-0412-7

**Published:** 2019-02-06

**Authors:** Connie Hoe, Daniela C. Rodriguez, Yeşim Üzümcüoğlu, Adnan A. Hyder

**Affiliations:** 1 Department of International Health, Johns Hopkins University Bloomberg School of Public Health, Baltimore, MD 21205 United States of America; 2 Department of Psychology, Middle East Technical University, Ankara, Turkey

**Keywords:** Agenda-setting, Health policy, Road safety, Tobacco control, Turkey

## Abstract

**Background:**

Tobacco use and road traffic injuries are major public health problems in Turkey. During the last decade, the former issue received political priority in the country, while the latter did not despite the immense health and economic burden that road traffic injuries pose on the Turkish population. Political priority can facilitate the attainment of public health goals. Unfortunately, however, limited cross-case analyses exist to help us understand why it emerges for certain public health issues but fails to develop for others in low- and middle-income countries.

**Methods:**

This study utilised Kingdon’s Multiple Streams Framework to explore the political priority development process in Turkey. A cross-case analysis was conducted, using data gathered from three different sources, namely key informant interviews (*n* = 42), documents (*n* = 307) and online self-administered surveys (*n* = 153). The Wilcoxon–Mann–Whitney test was also employed to examine whether the relationships within the tobacco control and road safety networks differed significantly.

**Results:**

In Turkey, political priority emerges when four streams – problem, policy, political and global – converge while a policy window is open. While these findings are largely consistent with the Multiple Streams Framework, this study also shed light on (1) the need to consider global health treaties for urgent public health issues as these instruments can accentuate global norms and standards, (2) the disproportionate strength of the political stream, (3) the need to develop in-depth understanding of national political context, (4) the importance of fostering meaningful ties between global and domestic health networks, and (5) the need for policy network cohesion.

**Conclusions:**

Findings from this study can be used by advocates striving to promote public health issues in other similar contexts.

## Introduction

Tobacco use and road traffic injuries (RTIs) are major public health concerns in Turkey, despite the availability of cost-effective interventions. According to the Global Burden of Disease study, tobacco smoking was the second leading risk factor for disease burden in Turkey and RTIs were the eighth leading cause of death for all age groups and first for Turks between the ages 5 and 49 years [1]. Fortunately, both issues received political attention in the last decade. In 2007, former Prime Minister and current President Recep Tayyip Erdoğan proclaimed tobacco control to be “*as important as our counterterrorism struggle, which is ever on our agenda*” [2]. Likewise, in 2012, Erdoğan declared road safety to be the second most important problem in the country after terrorism [3]. Despite these seemingly similar starting points, what followed were two strikingly different trajectories. For tobacco control, the 100% smoke-free legislation was enacted in January 2008 about a month after Erdoğan’s speech. More than 4000 inspectors were subsequently trained to enforce the law [4]. This allowed Turkey to become the third country in the world, after Ireland and the United Kingdom, to have a comprehensive smoke-free law [5], which is significant given the perceived historical and cultural importance of tobacco to the country. In 2013, Turkey also became the first country in the world to implement all of WHO’s MPOWER (*M*onitor, *P*rotect, *O*ffer help, *W*arn, *E*nforce and *R*aise taxes) measures, which conform a package of evidence-based policies, including smoke-free policies, that was developed to help countries reverse the tobacco epidemic [6, 7]. Turkey’s efforts have also been lauded by the international community [8] and the country ranked fifth in Europe in 2013 by total Tobacco Control Scale score, which is used to quantify the implementation of tobacco control policies [9]. This is in contrast to road safety, where limited legislative and institutional action followed to “*lock in*” ([10], p. 4) Turkey’s response. What explains these divergent paths?

Political priority is widely recognised as a critical facilitating factor in attaining public health goals [10, 11]. Despite its stated importance, limited cross-case analyses have been undertaken to help us understand why political priority emerges for certain public health issues but fails to develop for others in low- and middle-income countries (LMICs). Existing studies that have employed such a cross-case study design have largely focused on the successes and failures of one public health issue in different LMICs using qualitative data [11–17], yet few have compared different public health issues in one LMIC [18, 19]. Moreover, although existing studies have increasingly recognised the importance of global allies [17, 20, 21], there remains a need to explore whether the quality of the linkages between global and domestic networks can serve as a facilitating factor in national political priority development [22].

The primary aim of this study is to understand why two high-burden public health issues received varying levels of prioritisation despite expressed concern from the head of government and the presence of cost-effective interventions. We accomplish this by using Kingdon’s Multiple Streams Framework [23] to comparatively examine how tobacco control became a political priority in Turkey between 2002 and 2014 while road safety did not during the same timeframe.

## Conceptual framework

The Multiple Streams Framework was developed by Kingdon to examine transportation and health policy-making in the United States [23]. It has since evolved into one of the most renown and popular frameworks for agenda-setting. Although researchers have used this framework to examine various types of policies in high-income countries, few have employed Multiple Streams to better understand public health policy-making in middle-income countries [24–27]; as a result, the applicability of Kingdon’s framework to these countries needs further exploration.

Kingdon argues that, for issues to emerge onto the decision agenda, three process streams need to first develop, namely (1) the problem stream, which concerns the process whereby a condition turns into a problem that draws the attention of policy-makers and can be facilitated by the presence of indicators and/or focusing events, (2) the political stream, related to favourable macro-level domestic conditions, and (3) the policy stream, which concerns the emergence of an acceptable solution. These three streams need to be skilfully joined by policy entrepreneurs while a window of opportunity otherwise known as a policy window is opened. Kingdon defined policy entrepreneurs as individuals who are willing “*to invest their resources – time, energy, reputation, and sometimes money*” ([23], p. 122) to promote a cause and solution. He also stressed the benefits of having a tightly knit policy network specifically for the development of the policy stream, highlighting that a disjointed network often results in policy fragmentation. To unravel this concept, this study borrowed from collaboration literature to further define policy network integration as shared beliefs, frequent communication and trust amongst the members working on a public health issue [28, 29].

Although Multiple Streams did not include global-level factors, existing studies have shown that national policy-making cannot be fully understood without taking into consideration the global dimension [11, 16, 17, 20, 22, 24, 30, 31]. However, it is important to note that these global-level factors may influence issues and countries differently; donor dependent countries, for example, might be more vulnerable as compared to non-donor dependent countries. Accordingly, this study sought to enhance the existing framework by incorporating a fourth global stream, which we have defined as the presence of a favourable global environment for the public health issue of interest [20].

## Methods

This study used a mixed-methods cross-case study design to understand how political priority for public health issues develops. Political priority, our outcome of interest, has been operationalised as (1) expressed commitment from high-level decision-makers, (2) institutional commitment and (3) budgetary commitment [32].

Turkey was chosen as the study site due to its middle-income status, unique success with tobacco control, and high-burden of tobacco use and road traffic injuries. Tobacco control (case 1) and road safety (case 2) were selected using theoretical replication, wherein cases “*predict contrasting results but for anticipatable reasons*”([33], p. 54) based on the conceptual framework. Tobacco control and road safety are multi-sectoral, high-burden public health problems in Turkey that received different levels of policy attention between 2002 and 2014. Both drew international support through the adoption of global policy instruments and donor funding at around the same time.

### Data collection

Data collection was carried out in Ankara, Turkey. For each case, data were gathered from three different sources – key informant interviews, document review and online surveys. Key informant interviews and document review were conducted in 2013 and between 2010 and 2014, respectively. The purpose of the qualitative component was to explore the process and determinants affecting each domain of the conceptual framework and new themes that had emerged. The quantitative survey was carried out between 2013 and 2014 to examine the characteristics and relationships of the actors working on tobacco control and/or road safety in Turkey.

Across the two cases, a total of 42 in-depth interviews were performed with 39 different key informants. These informants included governmental, non-governmental, university and international actors (Table 1) who were purposively sampled [34] based on the following criteria: (1) had played a significant role in tobacco control and/or road safety policy process in Turkey and/or (2) possessed extensive knowledge about one or both issues in Turkey (Table 2 shows the list of informants and corresponding organisational affiliation). Recorded interviews were transcribed (*n* = 26) and notes from unrecorded interviews (*n* = 16) were converted into textual form. All transcripts and notes were analysed using deductive and inductive coding [35, 36].

**Table 1:** Characteristics of key informants for tobacco control and road safety (*n* = 39)

Variable	Categories	N(%)
Public health issue	Tobacco control	14 (35.9%)
	Road safety	21 (53.8%)
	Both	4 (10.3%)
Sex	Male	24 (61.5%)
	Female	15 (38.5%)
Organisational affiliation	Governmental organisation	15 (38.5%)
	International organisations	9 (23.1%)
	Turkish civil society/University	15 (38.5%)

**Table 2:** Informant IDs and organisational affiliations

Organisational affiliation	Informant IDs
Governmental organisation	i1, i2, i3, i4, i20, i21, i22, i23, i24, i25, i27, i32, i33, i34, i35
International organisation	i13, i14, i15, i16, i17, i18, i19, i37, i39
Turkish civil society/University	i5, i6, i7, i8, i9, i10, i11, i12, i26, i28, i29, i30, i31, i36, i38

Documents were purposively sampled from key informants, databases, including PubMed, Scopus, Türk Tip Veri Tabani, Sosyal Bilimler Veri Tabani and Ulakbim, web searches through Google Search engine, and the websites of Hürriyet Daily News and Today’s Zaman – the two most widely circulated newspapers in Turkey. A total of 307 documents were reviewed, of which the majority were newspaper articles (70%) followed by documents from relevant organisations (20%), published literature (8%) and national laws/regulations (3%). Data pertaining to the domains of the conceptual framework, new themes that had emerged, timeline of events and relevant quotes from actors were extracted onto an excel spreadsheet.

The online self-administered survey, which comprised of 10 questions, explored the attributes of the respondents and their ratings of the relationships within the tobacco control and road safety networks. A total of 153 individuals participated across the two cases. As census-based sampling frames did not exist for both issues, two databases that included the names and contact information of all individuals who had played a role in tobacco control and/or road safety in Turkey in 2013 were constructed. All identified individuals (*n* = 476) were invited to participate using Qualtrics [37]. The majority of the respondents were between 31 and 60 years of age (85%), males (57%), held doctoral degrees (49%), and worked on road safety/tobacco control for more than 10 years (41%) (Table 3).

**Table 3:** Characteristics of survey respondents for tobacco control and road safety (*n* = 153)

Variable	Categories	Road safety *n* (%)	Tobacco control *n* (%)	Total *n* (%)
Age range (*n* = 153)	18 to 30	7 (7.6%)	6 (9.5%)	13 (8.5%)
	31 to 40	33 (35.9%)	11 (18.0%)	44 (28.8%)
	41 to 50	24 (26.1%)	27 (44.3%)	51 (33.3%)
	51 to 60	22 (23.9%)	13 (21.3%)	35 (22.9%)
	61+	6 (6.5%)	4 (6.6%)	10 (6.5%)
Sex (*n* = 152)	Male	65 (70.7%)	22 (36.7%)	87 (57.2%)
	Female	27 (29.4%)	38 (63.3%)	65 (42.8%)
Education (*n* = 152)	Primary education	0 (0.0%)	0 (0.0%)	0 (0.0%)
	High school	1 (1.1%)	0 (0.0%)	1 (0.7%)
	Associate’s/Bachelor’s	29 (31.5%)	7 (11.7%)	36 (23.7%)
	Master’s	27 (29.4%)	13 (21.7%)	40 (26.3%)
	Doctoral	35 (38.0%)	40 (66.7%)	75 (49.3%)
Years working on the public health issue (*n* = 153)	Less than 5	31 (35.9%)	22 (36.1%)	53 (34.6%)
	5 to 9	21 (22.8%)	17 (27.9%)	38 (24.8%)
	More than 10	40 (43.5%)	22 (36.1%)	62 (40.5%)
Organisational affiliation (*n* = 152)	Government/Public sector	37 (40.2%)	11 (18.3%)	48 (31.6%)
	Civil society – Turkey	11 (12.0%)	5 (8.3%)	16 (10.5%)
	Civil society – International	0 (0.0%)	2 (3.3%)	2 (1.3%)
	University/Academia	22 (23.9%)	41 (68.3%)	63 (41.5%)
	International multilateral or bilateral organisation	4 (4.4%)	1 (1.7%)	5 (3.3%)
	Private sector/Industry	17 (19.6%)	0 (0.0%)	17 (11.2%)
	Others	1 (1.1%)	0 (0.0%)	1 (0.7%)

### Cross-case analysis

Cross-case analysis was employed for the purpose of identifying similarities and differences between the two cases [33]. The three-steps recommended by Miles and Huberman [38] were used as guidelines to ensure that data were organised and compared in a systematic manner.

Data from the two cases were first summarised and displayed in a matrix by the domains of the conceptual framework, thereby allowing for the general comparison of common themes. Subsequently, data were partitioned by the streams (problem, policy, political and global streams) then clustered into categories that were mentioned to have either contributed or prevented the development of the respective streams using four matrices. To better understand the actors, data were also partitioned by the different types of actors mentioned in both cases (i.e. Prime Minister, NGOs, etc.), then clustered by their roles, strategies and influences on the streams. Only themes that cut across both cases were maintained.

To complement these findings, quantitative data analyses were also employed to examine the similarities and differences between the two public health networks. In order to test whether they differed significantly with regards to their ratings of the relationships within their respective networks, the non-parametric Wilcoxon–Mann–Whitney test [39] was employed. This test was chosen as the dependent variables (i.e. frequent communication, agreement on the same solutions) were ordinal and the independent variable comprised two independent groups.

To draw conclusions, a final matrix was constructed to tie together all the findings from the previous steps. Cases were arranged by the main outcome (expressed commitment, institutional commitment and budgetary commitment) and streams and actors that preceded each outcome were identified. To further facilitate conclusion drawing, flowcharts were used to illustrate the processes that led to political priority development for tobacco control and the processes that led to the failure of political priority development for road safety. The four rules of thumb put forth by Miles and Huberman [38], namely that the variables (1) were ordered temporally, (2) could possibly have direct connections with other variables, (3) were claimed by informants to have linkages to other variables, and (4) were consistent with existing research and theories, were used as guidelines for flowchart construction.

## Results

### Problem stream

Tobacco use turned from a condition into a problem of concern that captured the attention of government officials in the 1980s, when Turkey began the process of privatisation leading to the abolishment of its state-owned tobacco monopoly (TEKEL) and the arrival of multinational tobacco companies, which used aggressive marketing to successfully increase sales [6]. In 1988, the Ministry of Health commissioned the first tobacco prevalence study, unveiling that 44% of the population smoked [40]. These indicators alarmed anti-tobacco control advocates, who were then primarily physicians working both within and outside of the government; they viewed the situation in Turkey as a crisis that needed to be addressed [20]. After a failed attempt to introduce an anti-tobacco law in 1991, early activists formed the National Coalition on Tobacco or Health (SSUK). Members of SSUK worked adamantly with a group of concerned parliamentarians to eventually pass the first anti-tobacco law in 1996; this law, although not comprehensive, was a tremendous success, banning direct advertising and smoking in some public places [6, 41].

Since the 1990s, tobacco use remained a problem of concern due to the growing number of studies that continued to shed light on the severity of the problem. The 2008 WHO Global Tobacco Epidemic Report, for example, highlighted the fact that two-thirds of the world’s tobacco was consumed by 10 countries, including Turkey [42]. Government officials explained:

“*We are one of the countries that consume too much.* [There is] *the quote ‘smoke like a Turk’*.” – Government Official (i2)

SSUK, then led by a policy entrepreneur, Dr Elif Dağlı, also continued to draw attention to the problem by organising courses, workshops, meetings and national anti-tobacco congresses [20].

In contrast, the issue of road safety appeared to have waxed and waned on the government’s agenda starting from the 1920s (i28, i31) ([43]; Şener S, Global Road Safety Program in Turkey: Updates from WHO, unpublished). The construction and use of new roads in the 1950s, for example, led to an increase in deaths, illuminating the inadequacies of existing laws, and prompting governmental actors to draft better legislations. It was then that the Road Traffic Act 6085 was enacted ([43]; Şener S, Global Road Safety Program in Turkey: Updates from WHO, unpublished). Likewise, in the 1990s, road safety re-emerged as a pressing problem due to the deteriorating situation in the country as highlighted by available indicators. To remedy the situation, the 1983 law was radically amended, increasing fines, establishing several governance boards and mandating road safety education [44]. Turkey also received a loan from the World Bank to start the Road Improvement and Traffic Safety Project [45]. As a result of these efforts and secular trends seen with advanced economic development [46], the country witnessed a significant decline in road traffic-related deaths.

However, the problem stream did not transform for road safety within the same timeframe. While road traffic crash and injury rates per 100,000 population were increasing in the country, the rates and numbers of road traffic death were decreasing [47, 48], and thereby did not trigger the attention of high-level governmental actors. This decline was even highlighted in one of Erdoğan’s speeches:

“*The number of people who died at the scene in traffic accident has decreased to 3757 despite the fact the number of vehicles exceeded 17 million*.” [3]

Moreover, road safety NGOs were described by key informants as “*limited*” (i17, i28), “*not powerful*” (i26), “*passive and fragmented*” (i39), with “*few having international connections*” (i28) to the global networks of actors, including the Global Alliance of NGOs for Road Safety, which was established in 2011. Although these NGOs conducted awareness-raising activities, provided public education and participated in government meetings, they did not engage in advocacy in a similar manner as the SSUK did for tobacco control and suffered greater fragmentation. Key informants suspected that this difference might be due to the presence of an enemy in the case of tobacco control:

“*I think that road safety doesn’t have a natural enemy the way that tobacco control does so there isn’t, to a certain extent, the need for an adversarial relationship with the government.*” – International Actor (i17)

“*The big difference is because we* [tobacco control advocates] *have a big industry to fight. That means that if you’re not structured you die*” – International Actor (i14)

Several government organisations, such as the General Directorate of Security (GDS) and General Directorates of Highways, have been actively working on road safety; however, these directorates rank low in Turkey’s political hierarchy and, hence, do not have sufficient power to affect other organisations.

“*Right now, police try to do something but it doesn’t have the power to affect other organisations because of its status*” – Government Official (i27)

### Political stream

In 2002, the Justice and Development party emerged into power, opening a new chapter for the country. Otherwise known as AKP, this social conservative party with roots in a 1960s Islamic movement [49] won the first majority government since the 1980s, ending over a decade of coalition governance. More notably, AKP drew votes from both conservative and moderate voters [50], winning the parliamentary majority not only in 2002 but also in 2007 and 2011.

There were several characteristics of the party that facilitated the tobacco control movement, and, to a much lesser extent, the road safety movement, including (1) religiosity, (2) interest from party leaders, and (3) desire to join the European Union (2002–2007), which, in later years, transformed into the desire to gain global visibility.

Many key informants identified this government transition as an important opportunity for tobacco control since many Muslims consider tobacco use as either *haram* (forbidden) or reprehensible in Islam [51, 52]. Moreover, AKP was led by Erdoğan who, fortuitously, detested tobacco use and personally cared about the issue (i6, i7, i10, i11, i12, i13, i14, i18). In a country where political power had predominantly resided with the Prime Minister [49], garnering Erdoğan’s support was found to be vital to the political priority development process:

“*There is a formula to this but I’ll be very brief. The formula is you have powerful government and a tough prime minister. You have to get the Prime Minister’s support otherwise it would be impossible. This is most important.*” – Turkish Academic (i7)

Road safety, on the other hand, “*is not a religious issue, it’s not a political issue*” (i39). It did, however, have some relevance to the party’s transportation plans (i8, i29) [53]). After years of instability in the country, the leaders of AKP took on a series of reforms [54], including major efforts to improve transportation for the purpose of boosting the country’s economy. Road safety was considered a small part of this grander scheme (i8, i29) [53].

Despite this, however, only one aspect – drunk-driving – appeared to have inspired genuine interest from Erdoğan [3]. His emphasis on drunk-driving as the main cause of road traffic crashes in Turkey during the 2013 launch of the Decade of Action for Road Safety sparked scepticism amongst road safety supporters as existing data showed that speed rather than drunk-driving was the leading risk factor; this further fuelled the belief that he was prioritising Islamic-oriented issues (i28, i29, i30) [48].

AKP also had ambitious foreign policy goals – to lead Turkey into the EU [53], particularly during the first term (2002–2007), and in later years, for Turkey to become one of the most powerful nations in the world [55–57]. These desires benefitted both public health issues as the country strived to harmonise with EU laws (i21, i23, i38) [20, 58]. Key informants, however, explained that global momentum to prioritise tobacco control was much stronger than it was for road safety; country successes in tackling tobacco use, for example, were highlighted in international discussions, providing further incentives for high-level decision-makers to take action:

“*I’m just stressing it now and here. I think part of the priority given to tobacco control in Turkey is linked to political will to have international successes and visibility because there is a treaty. With road safety there isn’t exactly the same platform for international visibility of Turkey in the international forum*.” – International Actor (i14)

### Global stream

The global stream transformed for tobacco control with the advent of the first global health treaty adopted by WHO – the Framework Convention for Tobacco Control (FCTC) [59]. Global activists, who formalised into the Framework Convention Alliance (FCA) in 2003, played a key role in the negotiation process [60, 61]. It is important to note that SSUK is one of the 350 member organisations of the FCA [62] and many of its members are physicians who possessed strong ties to the global anti-tobacco networks (i5, i6, i8, i9, i10, i14, i17). Embracing these foreign activist networks allowed the coalition to join forces with global allies to pressure the government to act [63, 64] and to learn global evidence and best practices, facilitating the diffusion of worldwide knowledge into Turkey (i5, i6, i9, i12) [65]. A key informant highlighted that as a result of these links:

“*Capacity-building increased,* [we were] *sharing the expertise and knowledge.*” – Turkish Civil Society Actor (i6)

In 2004, Turkey ratified the FCTC, an event described by key informants as one of the “*two chances for this topic*” (i1).

“*The FCTC was an opportunity for them* [politicians] *and for us* [non-governmental organisation] *of course*.” – Turkish Civil Society Actor (i11)

Similarly, for road safety, this stream developed as concerns for the issue led to the first Global Ministerial Conference on Road Safety hosted by the Russian Federation in 2009 [66]. The result of this meeting was the Moscow Declaration, which urged for the United Nations General Assembly to declare 2011 to 2020 the Decade of Action for Road Safety [67]. Turkey’s Minister of Transportation and high-level delegates from the Ministry of Health and the GDS participated in this historic meeting and pledged to take action. Key informants highlighted the importance of this event for road safety stating that “*the decade of action got it* [road safety] *speed. Forced us* [government officials]” (i25).

“*So years passed by and all of sudden this subject has come up in the agenda. When? In 2009. Why? Because in 2009, Turkey has put its signature on a declaration. This is the Moscow Declaration of the Road Safety so they could start the Decade of Action. So now Turkey is internationally responsible to do something because it has the signature on the paper*.” – Turkish Civil Society Actor (i28)

Subsequently, in 2010, the UN announced the Decade and in 2011 the Global Alliance of NGOs for Road Safety was established. However, while both the FCTC and the Decade of Action for Road Safety were important, results highlighted the fact that they were not equally influential:

“*The Decade of Action it’s a bit like World No Tobacco Day. It’s the kind of things that are nice but a treaty is a legally binding obligation*.” – International Actor (i14)

“*It doesn't help that, contrary to tobacco control, we don't have standards, we have recommendations* [for road safety].” – International Actor (i20)

Both public health issues also drew the attention and interest of Bloomberg Philanthropies (BP). In 2006 and 2010, BP launched the Bloomberg Initiative to Reduce Tobacco Use and the Global Road Safety Programme, respectively. Since its inception, BP has invested almost a billion to address tobacco use in LMICs [68], of which more than 2 million has been contributed as grants to Turkish organisations combatting tobacco use [69]. Likewise, between 2010 and 2014, BP invested 125 million to improve road safety in 10 LMICs, including Turkey [52]. Results showed that the Bloomberg projects were significant, as they helped (1) draw attention to the respective public health issues, (2) provide resources, (3) introduce international actors into the country and (4) invite new actors into the movements.

“*The fact that a big name like Bloomberg actually said it’s an important topic actually made very quickly the sensitivity, the awareness of the project*” – International Actor (i14)

### Policy stream

For both cases, actors who had been actively involved started drafting national action plans for their respective issues following the signing of global policy instruments. In the case of tobacco control, anti-tobacco advocates, both within and outside of the government, were largely in agreement about the need for a 100% smoke-free legislation, as it is a cost-effective intervention that aligns with the FCTC [20]. Some key informants suspected that the AKP was supportive due to their Islamic roots and indicated that Erdoğan was partly “*acting for religious reasons*” (i11). International actors who joined the movement as a part of the Bloomberg Initiative to Reduce Tobacco Use also helped boost momentum by providing resources to facilitate the enactment and implementation of the 100% smoke-free legislation [69]. These actors worked together with national actors to ensure that the legislation was consistent with international standards and “*ready to go*” ([23], p. 131) and joined forces to inspire public support for the solution [6, 20]. An opinion poll, for example, showed that 85% of Turks were in favour of the law [70].

Three policy entrepreneurs from within and outside of the government – the Head of Health Commission to Parliament and representatives from the WHO and president of SSUK – were also present to build broad-based support for the policy solution; the first two actors, for example, sought support from politicians by approaching different political parties and explaining to them the reasoning behind this ban, highlighting that it is a WHO recommendation [20].

In contrast, the road safety network suffered fragmentation, specifically with regards to policy solutions (i8, i17, i23, i24, i25, i27, i28, i29, i30, i38, i39). Actors working on road safety in Turkey were described to be “*holding very strongly to their one area*” (i39). Additionally, some believed that the priorities of the Bloomberg Global Road Safety Programme (encouraging restraint use and reducing speed) did not completely align with the priorities of the domestic supporters, such the GDS, who were interested in modernising the traffic system (i23, i24, i39). What also differed between tobacco control and road safety was the absence of policy entrepreneurs in road safety who can “*soften up*” the network and build acceptance for a solution (i36, i38).

Survey results also highlighted fragmentation within the road safety network. Compared to road safety respondents, tobacco control respondents believed that a higher percentage of individuals from international and Turkish organisations working on the issue of tobacco control in Turkey agreed on the same solutions (*p* = 0.000), frequently communicated (*p* = 0.000), and could be relied on to do what they say they will do (*p* = 0.000) (Table 4).

**Table 4:** Comparison of respondent ratings of the tobacco control and road safety networks

	Tobacco control	Road safety	Mann–Whitney test
	Median (IQR)	Median (IQR)	*p* value
What percentage of these individuals frequently communicate with each other regarding tobacco control-/road safety-related issues?	40% (40%–70%)	30% (35%–60%)	0.000*
What percentage of these individuals agree on the same solutions for tobacco control/road safety?	70% (60%–90%)	50% (30%–70%)	0.000*
What percentage of these individuals believe in tobacco control/road safety solutions that are based on scientific evidence rather than their own personal ideas?	70% (60%–90%)	50% (40%–80%)	0.000*
What percentage of these individuals bring unique perspectives to tobacco control/road safety in Turkey?	50% (40%–80%)	30% (20%–90%)	0.000*
What percentage of these individuals can be relied on to do what they say they will do?	60% (60%–90%)	50% (30%–80%)	0.001*

*p < 0.05

Survey findings further shed light on the difference between the two networks with regards to members’ belief in evidence-based solutions. Compared to road safety respondents, tobacco control respondents believed that a higher percentage of individuals from international and Turkish organisations working on the issue of tobacco control in Turkey trust in solutions that are based on scientific evidence (*p* = 0.000) (Table 4).

“[In tobacco control] *opinions are not so much divergent based on evidence. We don’t have to change opinions. In this* [road safety] *meeting people talk about opinions. They don’t have data. They just have opinions.*” – Turkish Civil Society Actor (i5)

A key informant contributed this to the presence of the FCTC:

“*I think honestly the FCTC has been extremely structuring for the whole of the movement because, after that, we got rid of all the people who were defending school programmes that lead to nowhere that kind of thing*” – International Actor (i14)

Road safety also suffered a higher order systems fragmentation. Responsibilities for road safety were scattered across various governmental organisations (Ministry of Interior, Ministry of Transportation, Ministry of Health, Ministry of Education and local authorities) and no ministry claimed to be the sole leader. Moreover, there are currently four coordinating bodies (Supreme Council on Road Safety, the Traffic Safety Council, the Road Safety Strategy Coordination Board, and the Road Safety Platform), which some informants believed have contributed to rather than facilitated the coordination issues (i26, i39). Although many governmental organisations are also involved in tobacco control, the Ministry of Health has been the leader in tackling this public health problem. Moreover, the health minister, Dr Recep Akdağ, was lauded by informants for being “*popular*” (i15), “*dedicated*” (i4, i9) and “*capable of bringing different groups together*” (i7) during his 2002 to 2013 tenure.

### Policy window

The opening of a policy window provides an opportunity for advocates to push their public health issue onto the government’s agenda [23]. Key informants identified the government transition in 2002 and the ratification of the FCTC in 2004 as the two events that led to the opening of a policy window for tobacco control [20].

“*I think it was after this party came into power in 2002. And FCTC was in the agenda. So, this and developments in Turkey came together.*” – Turkish Academic (i11)

During this time, all four streams were fully developed and the three policy entrepreneurs were present to join the streams. While SSUK, led by Dağlı, worked with international actors to amplify pressure on the government, the Head of Health Commission to Parliament and the WHO representative met with the Prime Minister to convince him to launch the National Tobacco Control Programme and Action Plan. These entrepreneurs were able to access Erdoğan as one (Head of Health Commission) had personal ties to him. During the meeting, they also briefed the Prime Minister about the 100% smoke-free legislation that was in queue to be considered by parliament [20]. In December 2007, during the launch, Erdoğan made his first strong statement against tobacco use, stating that the 100% smoke-free law will pass in 2008 [2]. Within a month’s time, the ban was enacted in Turkey. Subsequently, the Ministry of Health sponsored mass media campaigns to prepare the public and inspectors were trained to enforce the ban (Table 5).

**Table 5:** Level of political priority given to tobacco control and road safety in Turkey, 2002–2014

	Tobacco control	Road safety
Expressed commitment	*Prime Minister expressed his concern at three public events* • 2007: Prime Minister Erdoğan declared tobacco control to be as important as the country’s counterterrorism efforts^a,b^ • 2010: Prime Minister Erdoğan explained that tobacco use has become more destructive than terrorism^c^; Erdoğan also said at another event that his government is determined to continue its battle against smoking^b^	*Prime Minister expressed his concern at one public event* • 2013: Prime Minister Erdoğan declared road safety to be the second most important problem in Turkey, the first being terrorism^a,d^
Institutional commitment	The government enacted legislations that are up to international standards • 2006: A circular from the Prime Ministry promulgated the National Tobacco Control Programme and Action Plan • 2008: Enacted the 100% Smoke-Free Legislation • 2013: Implemented all of WHO’s MPOWER measures	The government closed a loophole in a regulation but has yet to amend the larger piece of traffic legislation that would nullify the remaining exemptions and allow the changes to the regulation to be more sustainable. Consequently, no institutional commitments were made that would ‘lock in’ Turkey’s response to road safety. • 2012: A circular from the Prime Ministry promulgated the Road Traffic Safety Strategy and Action Plan and established the Road Safety Strategy Coordination Board • 2014: Regulation 150, which exempted commercial car drivers from wearing seatbelts was closed, although not entirely
Budgetary commitment	The government allocated resources for tobacco control particularly as it relates to implementation of the 100% smoke-free legislation • 2008: Seracettin Çom explained that the Prime Minister fully backed the tobacco control campaign and the Ministry does not have a budget limit for it^c^ • 2008: More than 4000 inspectors were trained to enforce the law; in 2010, this number increased to more than 6000 to ensure that enforcement did not fade • 2013: Government expenditure on tobacco control was US$1.3 million^e^	Although there has always been some public funding for traffic safety, the amount is limited^f^ and has not increased after the launch of the Decade of Action for Road safety in Turkey^a^ Moreover, the implementation of the National Road Safety Strategy is not funded by the government^a,g^

Definition of Political Priority from Shiffman [10] and Fox et al. [11]

^a^Key Informants

^b^Today’s Zaman News [81]

^c^Hürriyet Daily News [82]

^d^Prime Minister’s Speech, 2013 [83]

^e^WHO, 2013 [7]

^f^SweRoad, 2001 [84]

^g^ GSRRS, 2013 [71]

Unfortunately for road safety, there did not appear to be any major events within the problem or political streams that might have helped open a policy window for the issue. Although policy entrepreneurs were absent, several informants did credit two key actors who had personal ties to Erdoğan – Mr Ismail Baş (Deputy Director General of Traffic Services) and Dr Cevdet Erdöl – for convincing the Prime Minister to reconvene the Supreme Council on Road Safety in 2012 after over a decade of inactivity and to launch the Decade of Action for Road Safety in 2013, respectively (i24, i27, i36, i37, i39). Unfortunately, however, limited institutional and budgetary commitments followed. The 2013 Global Status Report on Road Safety, for example, revealed that the implementation of the National Road Safety Strategy was not funded by the government [71] (Table 5). Likewise, informants explained that:

“*There are different* [road safety] *orders going from government to 81 provinces without any additional budget*.” – International Actor (i37)

## Discussion

This study contributes to our understanding of why political priority emerges for some public health issues but fails to develop for others in Turkey. As demonstrated in the case of tobacco control, prioritisation occurs when all streams are transformed and joined by policy entrepreneurs. Without all of these pieces in place at the same time, it is unlikely that a public health problem will be prioritised, as seen in the case of road safety, where only one of the streams was fully developed and policy entrepreneurs were absent. This study also shed light on the influence of global politics on Turkish national policy-making, the disproportionate strength of the political stream and the importance of fostering strong links between global and domestic health networks.

For both tobacco control and road safety, a supportive global environment, through the presence of global policy instruments and donors, helped facilitate the prioritisation process, specifically by drawing attention to the problem. Political scientists have long argued that countries that “*aspire to belong to a normative community of nations*” ([31], p. 29) and/or are more integrated in global society tend to be more vulnerable to global pressure [72, 73]. This appeared to be the case for Turkey between 2002 and 2014, when the country’s aspiration to join the EU and, in later years, to achieve global prominence [55–57], allowed it to be more receptive to global norms. Yet, the strength of the global stream appeared to have differed between the two cases; global pressure to tackle tobacco use was much stronger than road safety due to the presence of a binding legal treaty and an active global tobacco control network. As seen in this study, the FCTC not only drew attention to tobacco use (problem stream), but also helped facilitate integration as advocates supported solutions that aligned with the treaty (policy stream). It was also considered one of the factors that opened the policy window. Gneiting [74] shed light on the importance of the development of the FCTC in legitimising the global network of advocates and their framing of the problem.

While a supportive global environment is critical, its effects are limited by domestic factors. In Turkey, the transformation of the political stream appeared to be vital and, in particular, garnering the interest of the Prime Minister was found to be of utmost importance. As such, it was serendipitous for the tobacco control movement that Erdoğan was a devout anti-tobacco advocate and that the Head of the Health Commission to Government had personal ties to him. Likewise, it was unfortunate for the road safety movement that only aspects of the issue inspired genuine interest from Erdoğan. The disproportionate strength of the political stream could be explained by the fact that modern day Turkey evolved from a strong state tradition [75], where the position of the Prime Minister is held, most commonly, by the leader of the winning political party. Rabbani and Baroi also alluded to the immense power held by the Prime Minister in Bangladesh, referring to this individual as the “*supreme policy decision maker*” ([76], p. 250) and emphasising that, if high-level politicians advocate for an issue, it is quickly prioritised.

Although Turkey’s policy-making process appears to be privileged, non-state actors can also be influential, particularly when they are integrated. Tobacco control advocates coalesced around the 100% smoke-free legislation, rallying in one voice. The movement also enjoyed higher levels of communication and trust. In contrast, the road safety network was mired in fragmentation, with supporters promoting different solutions. Shiffman and Ved [27] explained that politicians might doubt a solution’s effectiveness when there are disagreements within the network of supporters.

The varying levels of integration might, in part, be explained by the degree of interplay between the global and domestic networks of actors. As a member organisation of the FCA, SSUK is tightly knit to the global tobacco control network, which has been very successful at diffusing its public health frame, namely that the global tobacco industry is the vector of the disease [74]. This is in great contrast to road safety, where few actors possessed linkages to their global counterparts. Gneiting and Schmitz [30] also unveiled three unique characteristics of the global tobacco control network that sets it apart from other global health networks. These global activists are keenly skilled at recruiting members from LMICs, sustaining agreement on policy solutions and uniting evidence with advocacy. Indeed, the Turkish tobacco control network was found to be more receptive to evidence-based solutions when compared to road safety.

The varying levels of integration between tobacco control and road safety might also be explained by differences in issue framing in Turkey. The perception of a common, undeniable enemy – the tobacco industry – provoked strong feelings amongst anti-tobacco advocates and facilitated cohesion [77]; this is not the case for road safety in Turkey, where there is currently no perceived enemy. Keck and Sikkink explained that

“*Problems whose causes can be assigned to the deliberate (intentional) actions of identifiable individuals are amenable to advocacy network strategies in ways that problems whose causes are irredeemably structural are not*” ([31], p. 27).

Moreover, while smoking is usually to blame for the majority of diseases that are attributable to tobacco use [78], road traffic injuries can result from multiple risk factors (i.e. infrastructure, driver behaviour, vehicle conditions and/or post-crash care), enhancing the issue’s complexity. High-income countries, like Sweden, that have effectively tackled road traffic injuries refocused their attention on the system rather than on the individual; reframing the solution as a safe systems approach [79].

The scope of this study was limited to two public health issues in one country. As a result, researchers should be careful when transferring these findings to other contexts. Moreover, causal inference is limited without additional comparative cases; accordingly, the factors described can only be considered as potential contributing factors. The timeframe under detailed investigation was also limited to 2002 to 2014. Although the history of each case was examined to identify factors that might have contributed to recent events, this study did not place focus on the processes that led to policy changes prior to 2002 or beyond 2014. Importantly, Turkey and the AKP have changed dramatically after the study concluded in 2014. Turkey’s desire to join the EU, for example, appears to have diminished [80], and the 2017 referendum will expand presidential powers, shifting the country into a presidential system.

## Conclusions

In Turkey, a public health issue is more likely to attain political priority when four streams – problem, political, global, policy – develop and converge while a policy window is open. While the Multiple Streams Framework was useful in unveiling the chronological ordering of the processes that led to political prioritisation of public health issues, it was less useful for disentangling the role of actors, suggesting the need to draw on or consider other frameworks such as the Advocacy Coalition Framework.

This cross-case study also demonstrates the need to consider global health treaties for urgent public health issues as they can intensify pressure on member states to adhere to global norms and standards; this tactic may be particularly effective for countries aspiring for global visibility.

While global politics are important, its effects can be limited by domestic factors, underscoring the need to better understand national political context. In a strong state like Turkey, garnering the interest of the Prime Minister appeared to be most critical, highlighting the role that strong leaders can play in such contexts. Despite this, advocates can still be influential if they rally in one voice, have allies with access to decision-makers and are tightly linked to the global network of advocates. As seen in the case of tobacco control, these connections helped foster cohesion, increasing global and domestic pressure on the Turkish government. This study attempts to quantify the level of cohesion. However, given that quantitative studies have rarely been conducted to compare the level of cohesion across different networks involved in political priority development, more studies will be required to answer the questions of how much cohesion is enough and the aspects of cohesion that are most important. As seen in Table 4, compared to road safety respondents, tobacco control respondents believed that a higher percentage of individuals from international and Turkish organisations working on the issue of tobacco control in Turkey frequently communicated with each other (*p* = 0.000); however, the difference is fairly small (40% vs. 30%), which could suggest that there are aspects of cohesion that may be more important (e.g. the network’s perception of cohesion and/or agreement on the same solution).

The strong link between the global and national network of advocates also helped diffuse effective framing strategies. For tobacco control, the portrayal of the tobacco industry as the enemy helped galvanise advocates, suggesting the need for advocates of other public health issues, like road safety, to devise effective frames that would evoke deeply held values to mobilise stakeholders and foster cohesion.

This study adds to the global health agenda-setting literature by illustrating how quality linkages between global and national networks can enhance effectiveness at the national level. Findings suggest the need for networks like the Global Alliance of NGOs for Road Safety to develop strong ties with national health networks in order to strengthen their agenda-setting power. Given the importance of the interplay between the global and national networks of actors, future research should also explore the structures and patterns of relationships that allow these global and national health networks to be most effective.

## References

[1] Global Burden of Disease 2013. http://www.healthdata.org/gbd. Accessed 1 Sep 2016.

[2] Erdoğan PM. Smoking more Dangerous than Terrorism. Today’s Zaman. 2007. http://www.todayszaman.com/home. Accessed 15 Dec 2014.

[3] Erdoğan RT (2013). Decade of Action for Road Safety.

[4] 4000 New Inspectors to be Hired to Enforce Smoking Ban. Today’s Zaman, Oct 25, 2009. http://www.todayszaman.com/home. Accessed 15 Dec 2014.

[5] Bilir N (2017). Successes and challenges in tobacco control – Turkish experience of 20 years. Eurasian J Pulmonol..

[6] Bilir N, Ozcebe H, Erguder T, Mauer-Stender K (2012). Tobacco Control in Turkey: Story of Commitment and Leadership.

[7] World Health Organization (2013). WHO Report on the Global Tobacco Epidemic 2013.

[8] Devi S (2012). Turkey wins plaudits for tobacco control. Lancet..

[9] Joossens L, Raw M. The Tobacco Control Scale 2016 in Europe. 2016. https://www.cancer.be/sites/default/files/tobacco_control_scale.pdf. Accessed 10 Dec 2018.

[10] Fox A, Goldberg A, Gore R, Barnighausen T (2011). Conceptual and methodological challenges to measuring political commitment to respond to HIV. J Int AIDS Soc..

[11] Shiffman J (2007). Generating political priority for maternal mortality reduction in 5 developing countries. Am J Public Health..

[12] Lee K, Walt G (1995). Linking national and global population agendas: case studies from eight developing countries. Third World Q..

[13] Munira S, Fritzen S (2007). What influences government adoption of vaccines in developing countries? A policy process analysis. Soc Sci Med..

[14] Omar AH, Ashawesh K (2008). Road safety: a call for action. Libyan J Med..

[15] Pelletier D, Menon P, Ngo T, Frongillo E, Frongillo D (2011). The nutrition policy process: the role of strategic capacity in advancing national nutrition agendas. Food Nutr Bull..

[16] Pelletier D, Frongillo E, Gervais S, Hoey L, Menon P, Ngo T, Stoltzfus R, Ahmed A, Ahmed T (2012). Nutrition agenda setting, policy formulation, and implementation: lessons from the mainstreaming nutrition initiative. Health Policy Plan..

[17] Smith SL, Shiffman J, Kazembe A (2014). Generating political priority for newborn survival in three low-income countries. Glob Public Health..

[18] Caceres C, Cueto M, Palomino N (2008). Policies around sexual and reproductive health and rights in Peru: conflict, biases and silence. Glob Public Health..

[19] Reichenbach L (2002). The politics of priority setting for reproductive health: breast and cervical cancer in Ghana. Reprod Health Matters..

[20] Hoe C, Rodriguez D, Üzümcüoğlu Y, Hyder AA (2016). “Quitting like a Turk:” How political priority developed for tobacco control in Turkey. Soc Sci Med.

[21] Tantivess S, Walt G (2008). The role of state and non-state actors in the policy process: The contribution of policy networks to the scale-up of antiretroviral therapy in Thailand. Health Policy Plan..

[22] Shiffman J, Schmitz H, Berlan D, Smith S, Quissell K, Gneiting U, Pelletier D (2016). The emergence and effectiveness of global health networks: findings and future research. Health Policy Plan..

[23] Kingdon J (2011). Agendas, Alternatives, and Public Policies (2nd Edition).

[24] Balarajan Y (2014). Creating political priority for micronutrient deficiencies: a qualitative case study from Senegal. Br Med J Open..

[25] Daniels K, Lewin S, The Practice Policy Group (2008). Translating research into maternal health care policy: a qualitative case study of the use of evidence in policies for the treatment of eclampsia and pre-eclampsia in South Africa. Health Res Policy Syst.

[26] Jat T, Deo P, Goicolea I, Hurtig A, San Sebastian M (2013). The emergence of maternal health as a political priority in Madhya Pradesh, India: a qualitative study. BMC Pregnancy Childbirth.

[27] Shiffman J, Ved R (2007). The state of political priority for safe motherhood in India. Br J Obstet Gynaecol..

[28] Foster-Fishman P, Berkowitz S, Lounsbury D, Jacobson S, Allen N (2001). Building collaborative capacity in community coalitions: a review and integrative framework. Am J Community Psychol..

[29] Mattessich P, Murray-Close M, Monsey B (2001). Collaboration: What Makes it Work. A Review of Research Literature on Factors Influencing Successful Collaboration (2nd ed.).

[30] Gneiting U, Schmitz H (2016). Comparing global alcohol and tobacco control efforts: network formation and evolution in international health governance. Health Policy Plan..

[31] Keck M, Sikkink K (1998). Activists Beyond Borders.

[32] Fox A, Balarajan Y, Chen C, Reich M. Measuring Political Commitment and Opportunities to Advance Food and Nutrition Security: A Rapid Assessment Approach. UNICEF Nutrition Working Paper. New York: UNICEF and MDG Fund; 2013. 10.1093/heapol/czu035 24902883

[33] Yin R (2008). Case Study Research: Design and Methods.

[34] Schutt R (2009). Investigating the Social World: The Process and Practice of Research.

[35] Charmaz K (2006). Constructing Grounded Theory: A Practical Guide Through Qualitative Analysis.

[36] Crabtree B (1999). Doing Qualitative Research.

[37] Qualtrics. Qualtrics Survey Software. Version 2013. Utah: Provo; 2005. Retrieve from https://www.qualtrics.com

[38] Miles M, Huberman AM (1994). Qualitative Data Analysis: An Expanded Sourcebook (2nd Edition).

[39] Forthofer R, Lee ES, Hernandez M (2007). Biostatistics: A Guide to Design, Analysis and Discovery (2nd Edition).

[40] Ministry of Health (1998). Tobacco Use in Turkey: The PIAR Study.

[41] No. 4207 Preventing Harms of Tobacco Products. 2007. http://www.tobaccocontrollaws.org/files/live/Turkey/Turkey%20-%20Law%20No.%204207.pdf. Accessed 14 Sept 2014.

[42] World Health Organization. WHO Report on the Global Tobacco Epidemic 2008. Geneva: WHO; 2008.

[43] Gökaşar I, Emir E. Changes in Traffic Safety Policies and Regulations in Turkey (1950–2010). 2011. http://www.iatss.or.jp/common/pdf/en/iatss/composition/7CountriesReport_en_04Turkey.pdf. Accessed 5 Jan 2015.

[44] Işıldar S. Road accidents in Turkey 1995–2004. IATSS Res. 2006;31(2):115–8.

[45] World Bank. Turkey Road Improvement and Traffic Safety Project. 1996. http://documents.worldbank.org/curated/en/1996/05/696491/turkey-road-improvement-traffic-safety-project. Accessed 20 Feb 2015.

[46] Bishai D, Quresh A, James P, Ghaffar A (2006). National road casualties and economic development. Health Econ..

[47] Turkish Statistical Institute (2008). Road Traffic Accident Statistics 2010.

[48] Turkish Statistical Institute (2013). Road Traffic accident statistics 2013.

[49] Finkel A (2012). Turkey: What Everyone Needs to Know.

[50] Kumbaracıbaşı A (2009). Turkish Politics and the Rise of the AKP: Dilemmas of Institutionalization and Leadership Strategy.

[51] Sucakli M, Ozer A, Celik M, Kahraman H, Ekerbicer H (2011). Religious Officials’ knowledge, attitude, and behavior towards smoking and the new tobacco law in Kahramanmaras, Turkey. BMC Public Health.

[52] World Health Organization. Bloomberg Philanthropies Global Road Safety Programme (2010–2014). 2014. http://www.who.int/violence_injury_prevention/road_traffic/countrywork/2014/en/. Accessed 1 Jul 2017.

[53] Party Programme. http://www.akparti.org.tr/english/akparti/parti-programme#bolum_. Accessed 1 Nov 2014.

[54] Karagöl ET. The Turkish Economy During the Justice and Development Party Decade. 2013. https://www.insightturkey.com/articles/the-turkish-economy-during-the-justice-and-development-party-decade. Accessed 18 Oct 2014.

[55] Adam L (2012). Turkey’s foreign policy in the AKP Era: has there been a shift in the axis?. Turk Policy Q.

[56] Cornell S (2012). What drives Turkish foreign policy?. Middle East Q..

[57] Stephens P. Turkey Turns East as Europe Clings to Past. 2009. https://www.ft.com/content/106a99e6-bf3d-11de-a696-00144feab49a. Accessed 5 Jan 2015.

[58] Bulut Y. Commission Approves Bill on Smoking Ban. http://www.todayszaman.com/national_commission-approves-bill-on-smoking-ban_126684.html. Accessed 1 Sep 2014.

[59] World Health Organization. Framework Convention for Tobacco Control. 2003. http://www.who.int/fctc/about/en/. Accessed 30 Sep 2014.

[60] Collin J (2004). Tobacco politics. Development..

[61] Wilkenfeld JP (2005). Saving the World from Big Tobacco: The Real Coalition of the Willing.

[62] Framework Convention Alliance. Membership Directory: Turkish National Coalition on Tobacco or Health. 2016. https://www.fctc.org/. Accessed 1 Oct 2016.

[63] Antismoking Campaign Gaining Momentum. Today’s Zaman, 4 May 2008. Retrieved from http://tobacco.cleartheair.org.hk/?p=425. Accessed 15 Dec 2014.

[64] Simpson D (1997). Bill gives hope that Turkey can fly. Tob Control..

[65] Framework Convention Alliance. Dr. Elif Dağlı – Turkish National Coalition on Tobacco or Health Chair. https://www.fctc.org/dr-elif-dagli-turkish-national-coalition-on-tobacco-or-health-chair/. Accessed 1 Oct 2016.

[66] Moscow Declaration. First Global Ministerial Conference on Road Safety: Time for Action. 2009. https://www.who.int/roadsafety/ministerial_conference/declaration_en.pdf. Accessed Jan 2019.

[67] United Nations. Letter dated 2 December 2009 from the Permanent Representative of the Russian Federation to the United Nations addressed to the Secretary-General. 2009.

[68] Bloomberg Philanthropies. Tobacco Control. 2017. https://www.bloomberg.org/program/public-health/tobacco-control/. Accessed 2 Jul 2017.

[69] Bloomberg Philanthropies. Bloomberg Initiative to Reduce Tobacco Use Grants Program. 2016. http://tobaccocontrolgrants.org/Pages/40/What-we-fund. Accessed 1 Sep 2016.

[70] Campaign for Tobacco Free Kids & Synovate’s Global Omnibus (2008). Memo: Turkey Survey Result.

[71] Global Status Reports on Road Safety. Country Profile: Turkey. 2013. https://www.who.int/violence_injury_prevention/road_safety_status/2013/en/. Accessed 15 Mar 2015.

[72] Meyer JW, Frank D, Hironaka A, Schofer E, Tuma N (1997). The Structuring of a World Environmental Regime, 1870–1990. Int Organ..

[73] Powell WW, DiMaggio PJ (1991). The New Institutionalism in Organizational Analysis.

[74] Gneiting U (2016). From global agenda-setting to domestic implementation: success and challenges of the global health network on tobacco control. Health Policy Plan..

[75] Dalay G. The Structural Roots of Turkey’s Power Struggle. 2014. http://www.gmfus.org/publications/structural-roots-turkey%E2%80%99s-power-struggle. Accessed 5 Jan 2015.

[76] Rabbani G, Baroi H (2012). Political priority and policy process: a recent example from Bangladesh. Asian Soc Sci..

[77] Weishaar H, Collin J, Amos A (2016). Tobacco control and health advocacy in the European Union: understanding effective coalition-building. Nicotine Tob Res..

[78] Wipfli H, Samet J (2015). Framing progress in global tobacco control to inform action on noncommunicable diseases. Health Aff..

[79] Bliss T, Breen J. Implementing the Recommendations of The World Report on Road Traffic Injury Prevention Country Guidelines for the Conduct of Road Safety Management Capacity Reviews and the Related Specification of Lead Agency Reforms, Investment Strategies and Safety Programs and Projects. Washington, DC: Global Road Safety Facility, World Bank; 2008.

[80] Sloat A. Here’s what Erdogan’s Referendum Means for Turkey, the EU, and the US. 2017. http://foreignpolicy.com/2017/04/17/heres-what-erdogans-referendum-means-for-turkey-the-eu-and-the-u-s/. Accessed 1 Jul 2017.

[81] World Health Organization. Turkey's Transformation. 2012. https://www.who.int/bulletin/volumes/90/6/12-030612/en/. Accessed 15 Jan 2019.

[82] Market for Products to Quit Smoking Growing in Turkey. Hürriyet Daily News. 21 May 2008. http://www.hurriyetdailynews.com/market-for-products-to-quit-smoking-growing-in-turkey.aspx?pageID%C2%BC438&amp;n%C2%BCmarket-for-products-to-quit-smoking-growing-in-turkey2008-05-21. Accessed 26 Apr 2016.

[83] United Nations Turkey Newsletter. Prime Minister Recep Tayyip Erdogan launched WHO “Decade of Action for Road Safety 2011-2020”. http://www.bmdergi.org/en/prime-minister-recep-tayyip-erdogan-launched-whodecade-of-action-for-road-safety-2011-2020/. Accessed 15 Jan 2019.

[84] SweRoad. National traffic Safety Program for Turkey. Solna: SweRoad; 2001.

